# Consensus statement from 2^nd^ International Conference on Controversies in Vitamin D

**DOI:** 10.1007/s11154-019-09532-w

**Published:** 2020-03-17

**Authors:** A. Giustina, R. A. Adler, N. Binkley, J. Bollerslev, R. Bouillon, B. Dawson-Hughes, P. R. Ebeling, D. Feldman, A. M. Formenti, M. Lazaretti-Castro, C. Marcocci, R. Rizzoli, C. T. Sempos, J. P. Bilezikian

**Affiliations:** 1 Chair of Endocrinology, School of Medicine, Vita-Salute San Raffaele University, Milan, Italy; 2 Division of Endocrinology, IRCCS San Raffaele Scientific Institute, Milan, Italy; 3 McGuire Veterans Affairs Medical Center and Virginia Commonwealth University School of Medicine, Richmond, VA USA; 4 Osteoporosis Clinical Research Program and Institute on Aging, University of Wisconsin-Madison, Madison, WI USA; 5 Section of Specialized Endocrinology, Department of Endocrinology, Oslo University Hospital, Rikshospitalet, Oslo, Norway; 6 Faculty of Medicine, University of Oslo, Oslo, Norway; 7 Laboratory of Clinical and Experimental Endocrinology, Department of Chronic Diseases, Metabolism and Ageing, Leuven, KU Belgium; 8 Jean Mayer USDA Human Nutrition Research Center on Aging, Tufts University, Boston, MA USA; 9 Department of Medicine, School of Clinical Sciences, Monash University, Clayton, VIC Australia; 10 Department of Medicine, Endocrinology Division, Stanford University School of Medicine, Stanford, CA USA; 11 Division of Endocrinology, Escola Paulista de Medicina – Universidade Federal de Sao Paulo (EPM-UNIFESP), Sao Paulo, Brazil; 12 Department of Clinical and Experimental Medicine, University of Pisa, Pisa, Italy; 13 Divison of Bone Diseases, Geneva University Hospitals and Faculty of Medicine, Geneva, Switzerland; 14 Vitamin D Standardization Program LLC, Havre de Grace, MD USA; 15 Department of Medicine, Endocrinology Division, College of Physicians and Surgeons, Columbia University, New York, NY USA

**Keywords:** Vitamin D, Osteoporosis, Fractures, Extra-skeletal effects, Food, Skin

## Abstract

The 2^nd^ International Conference on Controversies in Vitamin D was held in Monteriggioni (Siena), Italy, September 11-14, 2018. The aim of this meeting was to address ongoing controversies and timely topics in vitamin D research, to review available data related to these topics and controversies, to promote discussion to help resolve lingering issues and ultimately to suggest a research agenda to clarify areas of uncertainty. Several issues from the first conference, held in 2017, were revisited, such as assays used to determine serum 25-hydroxyvitamin D [25(OH)D] concentration, which remains a critical and controversial issue for defining vitamin D status. Definitions of vitamin D nutritional status (i.e. sufficiency, insufficiency and deficiency) were also revisited. New areas were reviewed, including vitamin D threshold values and how they should be defined in the context of specific diseases, sources of vitamin D and risk factors associated with vitamin D deficiency. Non-skeletal aspects related to vitamin D were also discussed, including the reproductive system, neurology, chronic kidney disease and falls. The therapeutic role of vitamin D and findings from recent clinical trials were also addressed. The topics were considered by 3 focus groups and divided into three main areas: 1) “Laboratory”: assays and threshold values to define vitamin D status; 2) “Clinical”: sources of vitamin D and risk factors and role of vitamin D in non-skeletal disease and 3) “Therapeutics”: controversial issues on observational studies and recent randomized controlled trials. In this report, we present a summary of our findings.

**Summary of the conference structure**

Conference was supported by an unrestricted grant from Abiogen, Italy - Program was developed independently by a Steering Committee (Chairs JPB and AG) Program was organized in three sessions (Diagnosis, Clinical aspects, Treatment). Each session was based on short introductory lectures and break out discussions. Three working groups on Diagnosis, Clinical aspects and Treatment drafted a document about the topics discussed in each session and these documents were examined in plenary discussion sections during which consensus on single point was reached. The writing group is responsible for correctly reflecting the contents and consensus reached during the meeting.

## Laboratory

### Vitamin D assay standardization: an update

Laboratory standardization of vitamin D is a necessary element in developing consensus regarding the serum 25-hydroxyvitamin D (25(OH)D) levels to define hypovitaminosis D [1–3]. Standardization is the process whereby all laboratories and assays are brought into alignment with the “true concentration” based on gold standard reference measurement procedures and certified reference materials [4–6]. Failure to utilize standardized 25(OH)D data is a major contributor to confusion surrounding vitamin D status [7].

Unfortunately, the vast majority of studies published to date include unstandardized 25(OH)D data. Despite the existence of performance testing/external quality assessment schemes (PT/EQA) - e.g. The Vitamin D External Quality Assessment Scheme (DEQAS)- there are still issues that need to be addressed. It is only since the development of certified 25(OH)D reference measurement procedures [e.g. US National Institute for Standards and Technology (NIST) [8]] and the introduction of The Vitamin D Standardization Program (VDSP) has it been possible to evaluate assay variation in an unbiased way [9]. This led to converting DEQAS [10] to an accuracy-based survey where standardized target values were assigned to serum samples using NIST reference measurement procedures [10]. Recent data from DEQAS have shown that such unstandardized assays are subject to significant assay variation over time [11, 12].

It has recently been shown that assays with or without standardization can lead to radically different results in individual studies [13]. In contrast, some studies in which assays were well calibrated originally, retrospective or after the fact, standardization had only a small effect. Good examples of these are the Canadian Health Measures Survey [14] and three German national surveys [15] which used the DiaSorin Liaison assay. In the three German surveys, the prevalence of 25(OH)D <30 nmol/L fell from 24% to 16%, 30% to 15% and 27% to 12.5%, respectively, after VDSP standardization. These results were to be expected based upon DEQAS data that previously showed that the DiaSorin Liaison assay reads low. However, in the Canadian Health Measures Survey, the prevalence of levels <30 nmol/L did not change after standardization [14]. These results lead to two conclusions: 1) the same commercial assay can have very different results depending on the laboratory and 2) because of the very recent development of the NIST reference measurement procedure and the VDSP, and retrospective standardization, it is unclear which data collected in the past were properly calibrated. Therefore, it is important to standardize all national surveys and 25(OH)D results from key studies.

Meta-analyses can suffer the same problem. However, little effort has been made to use only standardized data in meta-analyses. In the one example where standardized data were included, it was falsely claimed that standardization was not important. In an essay by Cashman et al., new results from a previously published paper [16] were included to evaluate the vitamin D intake necessary to meet the needs of 97.5% of the population to reach a 25(OH)D concentration of 50 nmol/L. In the re-analysis, it was reported that the value was 28.8 μg/day after VDSP assay standardization and 28.4 μg/day using unstandardized 25(OH)D data [17]. There are two possible explanations for the results: 1) the originally measurements correctly calibrated to VDSP guidelines or 2) this was the result of a series of errors cancelling each other out.

Related to assay standardization there is another potential problem. Certain immunoassays do not function properly in specific physiological/pathophysiological states. For example, due to high vitamin D binding protein concentrations some immunoassays yield inaccurate 25(OH)D results in pregnant women [18]. A recent paper illustrates the problems with using an untested immunoassay for measuring serum 25(OH)D in pregnant women [19]. The authors sought to assess the association between 25(OH)D measured at baseline (preconception), time to achieve pregnancy, at 8 weeks’ gestation and live birth outcomes. The interassay CV of the immunoassay was 15.8% at a mean concentration of 37.7 nmol/L, and 13.1% at 103.8 nmol/L for lyophilized manufacturer's controls and 17% for an in-house pooled serum control. The assay was not only poorly calibrated and its poor performance was not noted. Given the fact that bias can be associated with using immunoassays to measure 25(OH)D in pregnant women [20], the results cannot be easily interpreted. In these circumstances, 25(OH)D should have been measured at baseline (preconception) and at 8 weeks’ gestation with a VDSP standardized liquid chromatography-tandem mass spectrometry (LC-MS/MS). As a result, it is essential that researchers verify that their immunoassay of choice will function properly in the physiological/pathophysiological state under study by first determining that the immunoassay will yield results comparable to a VDSP certified standardized LC-MS/MS assay.

Vitamin D research data are used to develop government policy including public health/clinical guidelines. The utility and success of these guidelines depend on utilizing very accurate and precise 25(OH)D measurements. Only VDSP standardization can provide assurance that the data used to develop public policy are of the very highest quality because using unstandardized data can have long-terms consequences.

An example is the original source data used to define the 25(OH)D concentration at the lower limit of adequacy in the UK as 25 nmol/L [21]. The source was a 1976 paper with 9 cases of nutritional rickets and a range of 25(OH)D concentrations from 20 nmol/L to 54.9 nmol/L [22]. The performance characteristics of the assay were unclear and the cut-point of 10 ng/mL were not justified. Unfortunately, these concerns were not evident then, but government guidelines were set. Once government guidelines are set, they are very difficult to change. This has led to an impasse in which subsequent review panels have not been able to recommend a change in the "cut-points" despite the shortcomings of research database [19, 23, 24]. Thus, a lack of 25(OH)D standardization is at the root of the uncertainty regarding the definition of a vitamin D threshold for rickets, as well as for defining the entire clinical spectrum from deficiency to toxicity. Continuing to publish studies with flawed 25(OH)D data will perpetuate uncertainty about how to define vitamin D deficiency, adequacy, and toxicity.

There are two types of assay standardization: 1) prospective and 2) retrospective. Prospective standardization is the process whereby the initial measurements for a study are standardized using VDSP guidelines. Retrospective standardization of previously measured study samples is possible when appropriately stored serum samples are available [25–27]. In the discussion above, data from the Canadian Health Measures Surveys, the three German national surveys and the meta-analysis data cited by Cashman et al. were all retrospectively standardized. Three examples of prospectively standardized studies are NHANES 2011-2014 [27], Australian Health Survey [28], and a recent study on determinants of QuantiFERON-diagnosed tuberculosis infection in Mongolian schoolchildren [29].

### Threshold for defining vitamin D deficiency/insufficiency with current methods

An evidence-based consensus regarding the 25(OH)D concentration used to define hypovitaminosis D is needed. In the absence of compelling data, at this time, 25(OH)D values below 30 nmol/L should be considered to be associated with an increased risk of rickets/osteomalacia, whereas 25(OH)D concentrations between 50 and 125 nmol/L appear to be safe and sufficient in the general population for skeletal health [6, 30]. If we consider 30 nmol/L as the threshold for hypovitaminosis D, the question is whether we can apply this threshold using every current method (mostly automated immunoassays)? Comparisons among and between methods show a bias of several immunoassays compared to LC-MS/MS methodologies [31–34].

Within a given methodology, there are several possible causes for differences, such as lot-to-lot variation in manufacturer reagents or differences in subjects included in different studies. This last possibility leads to another important issue: if we can calculate a method-specific threshold, does this method-specific threshold hold for every group of subjects/patients? Several studies show this is probably not the case, as most immunoassays show matrix specific interference found in some physiological (e.g. pregnancy or ethnicity) and pathophysiological (e.g. intensive care unit, osteoporotic and haemodialysis patients) states. This latter point truly confounds one’s ability to compare levels across different clinical settings, even if the methodology is standardized and optimized.

In the recent method comparison studies described above, serum samples from different kinds of subjects were also used. Analysis of these separate groups shows rather large differences among the different groups of subjects.

Thus, if we consider 30 nmol/L as the threshold for hypovitaminosis D, we should recalculate this threshold for the various currently used automated immunoassays. This is complicated by matrix specific interference, as previously mentioned. All this reinforces the points made earlier: acquiring accurate and precise measurements of 25(OH)D in vitamin D research requires evidence that a selected immunoassay functions comparably to LC-MS/MS in the physiological/pathophysiological state under study and that the measurements be VDSP standardized.

### Threshold for defining vitamin D excess

All major agencies that promulgate nutritional guidelines have made recommendations, which also include tolerated upper levels of intake (TUL or UL). The current consensus for the UL for vitamin D in normal healthy individuals, represented by the Institute of Medicine 2011 recommendations [24], is 4,000 IU/day (100 μg/day). This value is based solely upon the consideration of vitamin D’s actions to regulate calcium and phosphate homeostasis. This UL may not be accurate in consideration of the putative non-calcemic functions of vitamin D. At steady state, an intake of 4000 IU/day corresponds to a mean serum 25(OH)D level of about 125 nmol/L in a normal healthy subject. It should be noted that the Endocrine Society guideline [35] for those requiring vitamin D therapy for various disease states recommends a UL of 10,000 IU/day (250 μg/day). A disease state, though, is not the same as a healthy state and thus one cannot forecast what the steady state level would be with levels of intake as high as 10,000 IU per day for a given disorder. At its extreme, someone who suffers with a malabsorption syndrome may require amounts even greater than 10,000 IU per day in order to maintain a reasonably normal level of 25(OH)D.

Authoritative agencies have concerns about vitamin D toxicity from long-term, moderate dosing (chronic toxicity) as well as short-term, high-dose therapy (acute toxicity). Consequently, the ULs and the corresponding serum 25(OH)D levels achieved have become more conservative [24].

Limited human studies involving acute toxicity, mainly from anecdotal reports regarding accidental overdosing with vitamin D3 [36, 37], suggest that doses of over 10,000 IU/day and serum 25(OH)D levels of around 250 nmol/L can be tolerated in the short term. These studies usually involve minimal data prior to the appearance of the toxicity and consequently, the events which trigger hypercalcemia remain obscure. Of course, well-documented human trials examining the effect of excessive doses of vitamin D are ethically impossible so that there is a paucity of evidence regarding the exact mechanism by which vitamin D intake causes toxicity. From published reports, though, it is clear that vitamin D toxicity can occur via ingestion of over the counter products such as Soladex, that contains over 800,000 IU of vitamin D per dose [38]. Genetic disorders in which vitamin D is not normally metabolized can lead to excessive amounts of 1,25(OH)2D, despite normal intake of vitamin D [39].

Physicians providing vitamin D to patients should be reminded that there are genetic and acquired diseases involving dysregulated vitamin D metabolism that can alter vitamin D intake requirements [34]. In addition to malabsorption syndromes noted above, obesity can be associated with vitamin D being sequestered in fat tissue. In chronic kidney disease (CKD), impaired activation of vitamin D presents the need to provide active metabolites. Similarly, in advanced liver disease, inability to hydroxylate cholecalciferol requires vitamin D forms that are active. Genetic disorders in which vitamin D is not normally metabolized can lead not only to excessive production of active vitamin D and hypercalcemia or kidney stones or nephrocalcinosis [39, 40], as noted above, but also to excessive metabolism of vitamin D by activation of cytochrome P450 3A4 (CYP3A4) resulting in rickets or osteomalacia [39, 41–43].

With these many uncertainties, it is difficult for agencies to recommend a specific IU, but most have settled on 4000 IU/day as a safe upper intake level for vitamin D [24]. However, the range of vitamin D intake of between 4,000-10,000 IU/day [24, 35] will probably remain as a useful safe buffer zone that physicians can use in the short term and will not result in serum 25(OH)D levels of over 250 nmol/L.

Again, this advice relates to normal, healthy individuals. To apply this advice to some of the conditions described in this section will lead to gross undertreatment of patients who need considerably higher daily doses of vitamin D. This standard advice also does not pertain to individuals who are grossly vitamin D deficient, in whom there may well be an indication to increase levels quickly by using higher doses in the short term.

### Should we still be prescribing ergocalciferol?

The two parent forms of vitamin D, namely ergocalciferol [vitamin D2 or 25(OH)D2] and cholecalciferol [vitamin D3 or 25(OH)D3] are widely available and used commonly. In the United States, there is no over-the-counter form of vitamin D2 or D3 in the 50,000 IU dosage; in the prescription form at that dose, only vitamin D2 is available in the USA [35]. There are methodological challenges to the measurement of 25(OH)D when immunoassays are used related to the coexistence of both circulating 25(OH)D2 and 25(OH)D3. Specifically, immunoassay antibodies may not detect 25(OH)D2 and 25(OH)D3 equally, and the proprietary releasing agent in these automated assays to free 25(OH)D from vitamin D binding protein may not liberate 25(OH)D3 and 25(OH)D2 equally.

To explore these considerations, a pilot study was performed in which residual plasma was collected from routine clinical laboratories [44]. Sample pools containing predominantly 25(OH)D3 from 20-255 nmol/L and 10 with 25(OH)D2 from 35-197.5 nmol/L were prepared. Eight independent laboratories analysed these pools using their routine 25(OH)D immunoassays. Data for five FDA-approved automated methods were compared to total 25(OH)D determined by an High Performance Liquid Chromatography (HPLC) assay calibrated to NIST assigned values. These pooled specimen results provided regression equations for total 25(OH)D using the various immunoassay methods. When the results are considered based on the primary form present, i.e. 25(OH)D3 or 25(OH)D2, agreement with HPLC results dramatically improved for 25(OH)D3.

To improve immunoassay accuracy, we suggest focusing on 25(OH)D3 to harmonize commercial immunoassays, since 25(OH)D3 is naturally produced and is the dominant supplement form worldwide.

In addition to causing potentially insurmountable immunoassay challenges, we believe that high-dose ergocalciferol is not the best clinical approach to vitamin D repletion. Cholecalciferol supplements (even 50,000 IU) are widely available at low cost. Thus, we believe the common clinical practice of treating vitamin D deficiency by prescribing high-dose ergocalciferol is no longer best clinical practice. Therefore, we suggest that the first-line of treatment is cholecalciferol where possible and that ergocalciferol only is used for vegans and in other patients opposed to using cholecalciferol. However, it must be remembered that when ergocalciferol is used monitoring of 25(OH)D levels will require measurements made with VDSP-standardized HPLC or LC-MS/MS assays.

### Age as a specific threshold determinant in the general population

It is well established that advancing age reduces skin capability to synthesize pre-vitamin D3 [45]. Moreover, the prevalence of skin cancer in older adults has reached "epidemic" proportions with a resultant array of recommendations (including a Surgeon General's report) advising avoidance of skin exposure to the sun [46]. As such, it could be expected that older adults would have poorer vitamin D status. Indeed, higher 25(OH)D levels have been reported in children [47]. Moreover, the Institute of Medicine appears to have acknowledged this point establishing a Recommended Dietary Allowance (RDA) of 800 IU/day for those > age 70 years [48], higher than the amount recommended for younger populations (600 IU/day). Similarly, the International Osteoporosis Foundation recommends a higher average vitamin D intake of 800-1,000 IU for older adults [49].

These recommendations are predicated on the expectation that older adults are more likely to be vitamin D deficient. Some studies have reported lower circulating 25(OH)D levels with advancing age [49, 50]. For example, a meta-analysis of 33,000 subjects (using unstandardized 25(OH)D data) found those aged >75 years to have 25(OH)D values on average 9 nmol/L lower than those aged 65-75 years. However, a more robust approach using a representative sample (NHANES 2007-2010) and, importantly, standardized 25(OH)D data found no evidence to support lower 25(OH)D concentration in older adults overall or when stratified by race/ethnicity [47]. Indeed, those age >60 years had higher 25(OH)D values than those aged 40-59 years in the entire cohort, including Hispanics and non-Hispanic blacks. Consistent with this, the prevalence of "low" vitamin D status (using 50 and 75 nmol/L as cut-off points) was numerically lower in those aged >60 years than among adults age 40-59 years among all race/ethnic groups [47]. Additionally, multiple studies find no effect of age on response of 25(OH)D to oral vitamin D supplementation [51].

Similar to NHANES, data from the national surveys in Finland, Ireland, Germany and Canada do not find dramatic differences in standardized serum 25(OH)D or vitamin D inadequacy prevalence (defined as a 25(OH)D < 50 nmol/L) based upon age [14, 15, 52, 53]. However, institutionalized older adults may be at higher risk for vitamin D deficiency, presumably due to limited sun exposure and inadequate supplementation [54, 55].

Thus, despite expectations that older individuals have lower levels of 25(OH)D on average, age as a specific determinant does not seem to be a key factor. It remains to be seen whether age could contribute to vitamin D status when other risk factors are also present.

**Consensus Statements:**

1. Existing data are insufficient to define “low” or “high” vitamin D status thresholds with any degree of certainty because of the lack of standardized 25(OH)D measurements in vitamin D research.
2. The current approach to defining vitamin D status using circulating 25(OH)D concentration with standardized state-of-the-art methodology is recommended.
3. Due to assay variability, circulating “25(OH)D” as measured by the multitude of existing assays cannot simply be blindly pooled into meta-analyses. Meta-analyses should not be conducted including studies that use non-standardized assay methodology.
4. For research and for publication of data, 25(OH)D assays should demonstrate standardization or alignment with reference methodology along the lines proposed by the VDSP.
5. Laboratories should participate in a 25(OH)D accuracy-based proficiency testing program, e.g. DEQAS or College of American Pathologists (CAP).
6. Some documentation that the 25(OH)D assay methodology functions properly in the setting being studied, (e.g. pregnancy, hemodialysis) is needed. This can be accomplished by comparison of the assay method being used with a standardized method in the physiologic condition being studied. Given existing assay deficiencies assay manufacturers should develop assays that have comparable ability to accurately measure 25(OH)D2 and 25(OH)D3 in various clinical circumstances.
7. It seems reasonable to recommend that cholecalciferol rather than ergocalciferol is used for vitamin D supplementation for most people.
8. The risk of developing rickets/osteomalacia is increased at a 25(OH)D concentration of ≤ 30 nmol/L. This threshold may vary depending on other conditions such as calcium and phosphate nutrition, parathyroid hormone (PTH) levels, and season.
9. The 25(OH)D concentration ranges among normal subjects between approximately 50-125 nmol/L [56, 57]. With admitted uncertainty, an upper 25(OH)D threshold of 125 nmol/L is advisable.

**Research agenda**

1. Determine whether threshold values for 25(OH)D (both low or high) are applicable under various clinical conditions.
2. Develop a reference measurement procedure and reference materials for free 25(OH)D assessment.
3. Determine the added value of free 25(OH)D measurements in the assessment of vitamin D status.
4. Evaluate the utility of the vitamin D metabolome in various physiologic and pathologic conditions.
5. Determine variables that affect the utilization (absorption/metabolism/biologic action/transport/storage) of vitamin D.
6. Determine whether vitamin D status (i.e. “sufficient”, “insufficient” and “deficient” levels) influences supplementation adherence/persistence and outcome.

## Clinical

### Sources of vitamin D

#### Food

Vitamin D deficiency and associated health risks are problems that still need to be managed and addressed worldwide. Generally, dietary intake recommendations are not met. The calculation of dietary intake is based on a combination of content of vitamin D in our food and information on the amount of food consumed.

Analytical data for vitamin D in food samples are no better than the sampling strategy and the performance of the analytical methods. Due to the development of simpler and cheaper chemical analyses of vitamin D, information on vitamin D content in food has markedly increased in the the last 5-10 years. In food, vitamin D activity derives from the parent forms vitamin D3 and vitamin D2, and the corresponding hydroxylated forms 25(OH)D3 and 25(OH)D2.

The best available vitamin D sources are cod liver oil (90-250 μg/100g) and fatty fish, e.g. aquaculture salmon (6-10 μg/100g) and wild mackerel (5-8 μg/100g) [58–61]. But as dietary intake depends on the amount consumed, food products with lower content also play a role. Thus, vitamin D3 and 25(OH)D3 are found in fish, eggs, meat, and dairy products [58]; vitamin D2 is found in wild mushrooms or cultivated mushroom exposed to ultraviolet B (UVB) rays, whereas beef and dairy products contain vitamin D2 and 25(OH)D2 [62–64].

To calculate total vitamin D activity in food, conversion factors between the different vitamin D forms are essential [60]. The contribution to vitamin D activity from 25(OH)D3 compared to 25(OH)D2 has been assessed in human intervention studies [61–63]. Studies that have compared the effect of dietary intake of vitamin D3 and vitamin D2 on vitamin D status have been evaluated in a systematic review and meta-analysis [64]. The overall conclusion is that when vitamin D is administered once or as a monthly bolus, vitamin D3 is superior to vitamin D2 in increasing vitamin D levels, whereas no difference in vitamin D status is observed if vitamin D2 and vitamin D3 are administered on a daily basis [64]. The only human intervention study performing a randomized cross-over design examining the difference in vitamin D status by administration of daily vitamin D3 and vitamin D2 found vitamin D3 to be superior in maintaining vitamin D status [63].

Foods can be fortified with vitamin D and, in this setting, would be referred to as bio-fortified. In the European Union, the beneficial effects of this strategy are limited due to a restriction of the maximum content of vitamin D in feed for livestock [65]. However, apart from this limitation, it is still possible to increase the content of vitamin D in food of animal origin. In Denmark, for example, the current recommendation is 400 IU/kg feed to slaughter pigs. If this limit was increased to the maximum EU level of 2,000 IU/kg feed, the content of vitamin D in the food product would increase by a factor of 3 [66]. In eggs, vitamin D may be increased 10-fold compared with the current maximum level, without harming hens. In this example, one egg could contain the entire day’s nutritional recommendation [67].

Intervention studies have shown the possibility of increasing dietary intake with dietary supplements, enriched food and bio-fortified food. Some countries have chosen to introduce mandatory fortification (e.g. Finland), while other countries’ foods are enriched, but not through legislation (e.g. USA). Other countries continue to be engaged in a debate as to what strategy is best for their needs.

#### Skin

Solar UVB (~295-315 nm) light is the main source of vitamin D for most human beings. It is well known that at a given latitude individuals with darker skin have poorer vitamin status [25(OH)D3] than those with lighter skin. Latitude is a determining factor for the extent of UVB exposure. Differences in exposure to this wavelength of light are usually attributed to the photoprotective (i.e. inhibitory) properties of melanin. However, studies investigating the role of melanin are inconsistent. The impact of melanin on vitamin D status was compared across a range of skin types (white to black) after whole body exposure with two different solar-range Ultraviolet Radiation (UVR) spectra. UVR exposures were fixed across skin types for a given UVR source and were fixed to be sub-erythemal; thus, the only clear variable was skin color. Repeated exposures were given at 3-4 day intervals and blood samples were taken before irradiation and after the final irradiation for the assessment of 25(OH)D3. The UVR dose response curves for 25(OH)D3 were linear and the ratio of the slopes of black and white skins were compared to estimate a melanin protection factor, which was <1.5 with both UVR sources. This result shows modest inhibition of vitamin D production by melanin, but it is insufficient to explain the epidemiological differences in different skin types.

Sunscreens are effective because they attenuate solar UVR. Their use can inhibit cyclobutane pyrimidine dimers (CPDs) [68], sunburn and skin cancer [69]. The sun protection factor (SPF) of a sunscreen is primarily an index of its ability to attenuate UVB [70]. Thus, in theory, sunscreen use would be expected to inhibit the production of vitamin D. However, there are conflicting views about whether this is the case in practice because sunscreens are typically used sub-optimally [71, 72]. This could be due, at least in part, to the need to reapply such screens at regular intervals to maintain the protective effect or incomplete skin covering with sunscreen.

By definition, the higher the UVA protection factor (UVA-PF) for a given SPF, the lower the UVB protection. Two SPF = 15 sunscreens used correctly during a week of perfect weather with a maximum daily UV index (UVI) of 9 (deemed very high) were compared with each other. In study by Petersen and colleagues [73], participants included one group with a low UVA-PF (n=20) and the other with a high UVA-PF (n=20), and instructed on how to use sunscreens to achieve the labelled SPF (i.e. apply 2mg/cm^2^ skin). Another group (n=22) was told to bring their own sunscreens and use as they do typically, which might be expected to be ~0.8mg/cm^2^ [73]. Participants in the typical use group had sunburn of 5 in exposed body sites. Sunburn was not observed in the sunscreen intervention groups. All groups had a highly significant increase in serum 25(OH)D3 at the end of the test period. The increase in the typical use group was greater than both sunscreen intervention groups, and the increase in the high UVA-PF group was greater than in the low UVA-PF group. These data show that even when sunscreens are used optimally to prevent sunburn under very high UVI conditions, they permit skin synthesis of vitamin D.

**Consensus Statements:**

1. The most recent analysis of food content of vitamin D confirms a low vitamin D content in non-fortified food supplies.
2. There appear to be differences in the bioavailability and perhaps biological effects between 25(OH)D3 and 25(OH)D2 under certain circumstances.
3. Vitamin D or 25(OH)D3 can be added to the food supply by feeding animals with fortified feed.
4. The most recent data on exposure to very high natural sunlight exposure have led to uncertainty about how much vitamin D3 can be produced after sun exposure.
5. Individuals with dark skin have the capacity to produce vitamin D3 to a greater extent than previously assumed.
6. Real-life usage of sunscreen does not markedly affect production of vitamin D3 in the skin.

**Research agenda**

1. Compare 25(OH)D3/vitamin D contents in foods from different areas of the world.
2. Establish in more detail how fortified feed to animals is transformed into higher vitamin D in the food product and ultimately in the human circulation.
3. Determine with greater certainty how much vitamin D3 can be produced in the skin under different conditions of sun exposure.
4. Determine to what extent and how the effects of UV light on skin damage and vitamin D3 synthesis can be differentiated from each other.
5. Determine whether there are other effects of UV light on the skin, and the extent to which they are related to vitamin D3.

### Risk factors for vitamin D Deficiency

#### Glucocorticoids

Patients exposed to glucocorticoid excess have a two-fold higher risk of vitamin D deficiency compared to the general population, that is most likely related to the underlying disease and the direct effects of glucocorticoids on vitamin D metabolism [74].

It is still unclear whether these effects on vitamin D metabolism may occur clinically, since serum calcitriol [1,25(OH)D] values are variable in patients with glucocorticoid-induced osteoporosis (GIO) [75]. Moreover, vitamin D receptor expression may be decreased by glucocorticoid excess in several tissues and cells, leading to a vitamin D resistant state [76, 77]. These actions of glucocorticoids on vitamin D metabolism and activity may be negatively synergized by the underlying disease for which glucocorticoid treatment is given [78]. Based on these pathophysiological concepts, the use of calcitriol or alfacalcidol has been proposed instead of cholecalciferol for the treatment of hypovitaminosis D in glucocorticoid-induced osteoporosis [79].

Randomized studies have demonstrated that combination therapy with calcium and vitamin D was shown to be more effective in preserving bone mineral density than either calcium or no treatment with a weighted mean difference of 2.6% and 2.5% at lumbar spine and forearm, respectively [80]. Moreover, the administration of both vitamin D and calcium is expected to potentiate the anti-fracture effects of bone-active drugs in glucocorticoid-induced osteoporosis, such as already demonstrated in post-menopausal osteoporosis [81].

Therefore, vitamin D and calcium are recommended in patients exposed to therapeutic amounts of glucocorticoids.

#### CKD

In patients with CKD, alteration in vitamin D metabolism plays a central role in the development of secondary hyperparathyroidism (SHPT), in addition to being associated with increased cardiovascular morbidity and mortality [82]. A hallmark of vitamin D insufficiency/deficiency is elevated levels of parathyroid hormone and consequently most guidelines on the use of vitamin D in CKD have largely been based on levels of PTH and calcium. UVB exposure is effective in increasing the serum levels of 25(OH)D even in patients with end-stage kidney disease on dialysis [83].

Since the late 7O's, native vitamin D and nonselective vitamin D receptor (VDR) activators have been used mainly for lowering of PTH levels [84, 85]. In the past two decades, selective VDR activators have gained recognition for their importance in the management of CKD-mineral and bone disorder (MBD) and as such are considered standard therapy in these patients. More recently, vitamin D deficiency has been linked to a whole host of diseases, some related to CKD, prompting further exploration of the mechanism of action of active vitamin D analogues and, consequently, their potential benefit in clinical trials. However, many open questions regarding the use of native vitamin D or VDR activators remain [86–89]. First, the Kidney Disease Outcomes Quality Initiative (K/DOQI) and Kidney Disease: Improving Global Outcomes (KDIGO) guidelines recommend testing for vitamin D insufficiency and deficiency in CKD patients, but no consensus on the definition of vitamin D insufficiency in CKD is currently available [90]. Second, using native vitamin D in patients with CKD remains heavily debated, particularly with regard to the choice of compound, when to treat, and the most suitable dose to administer with or without VDR activators [89].

Interestingly, 25(OH)D serum levels <37.4 nmol/L have been repeatedly reported in up to 50% of CKD 5 stage patients [91], while severe vitamin D deficiency seems to be present in approximately 95% of patients on hemodialysis [92]. The same findings have been reported for kidney transplant recipients, even in those with relatively well-preserved renal function [93]. Together with these findings, vitamin D deficiency has been associated with several complications of CKD, such as cardiovascular disease, anemia, proteinuria, progression of renal failure and disordered calcium metabolism [89, 94]. After kidney transplantation, low levels of 25(OH)D serum levels are the most important predictor of the sustained increase in PTH levels, which persists in up to 50% of patients even many years after surgery [93].

In a six-month prospective controlled study, Molina et al. [95] showed that cholecalciferol was able to reduce albuminuria even after controlling for a range of potential confounders. In a recent randomized, double-blind, placebo-controlled trial, cholecalciferol supplementation was associated with an improvement in vascular function in non-diabetic CKD 3-4 stage patients [96]. After renal transplantation, cholecalciferol administration has been found to significantly reduce PTH levels. Finally, a recent randomized, double-blind, placebo-controlled study from Yadav et al. [97], in which cholecalciferol was given to patients with CKD stages 3-4 before transplantation, PTH levels declined significantly. The authors also showed a substantial decrease in bone alkaline phosphatase activity and C-terminal cross-linked collagen type I telopeptide, strongly suggesting that the cholecalciferol­ induced decrease in PTH may reduce bone remodelling in these patients.

**Consensus Statements:**

1. Glucocorticoids are associated with vitamin D deficiency and/or resistance.
2. The pathophysiology appears to be multifactorial.
3. Vitamin D supplementation improves skeletal health in glucocorticoid-induced osteoporosis when combined with calcium.
4. The precise effects of native vitamin D compounds in the treatment of some very common complications of CKD are far from fully established. However, several recent findings seem to strongly support a beneficial role of such a therapy in this setting as well.
5. While awaiting more “solid” data, we can assume that oral vitamin D, such as cholecalciferol, can provide benefit in several clinical settings related to CKD.

### Vitamin D as a risk factor for skeletal health

Vitamin D is generally considered to be "good for bone" because it can prevent or cure nutritional rickets and osteomalacia [98]. This effect is largely mediated by increasing intestinal calcium absorption in the small intestine, primarily in the proximal jejunum. The genes and proteins involved include transient receptor potential cation channel subfamily V member 6 (TRPV6), calbindins and CaATPases as well as genes/proteins related to tight junctions (claudins) [99].

Other sets of data can give another impression, namely that vitamin D has no direct effect on bone, because high calcium intake (orally or intravenous) can normalize bone and growth plate structure in animals or patients with complete lack of vitamin D action (by VDR or CYP27B1 mutations) [100–102]. This conclusion is also supported by the absence of a clear or marked bone phenotype in cases of VDR deletion in osteoblasts, osteocytes or osteoclasts.

Finally, other data provide yet another scenario, namely that vitamin D can be "bad for bone" [98]. Indeed, in the case of severe calcium deficiency (or malabsorption of calcium) high serum concentrations of 1,25(OH)2D can stimulate bone resorption (RANKL-mediated) and directly inhibit bone mineral deposition (by increasing osteopontin and pyrophosphate concentrations) [103]. In addition, most cases of rickets due to phosphate deficiency are associated with high serum 1,25(OH)2D concentrations [104].

There is no unanimity about the possible role of other vitamin D metabolites apart from 1,25(OH)2D and its inactive precursor on bone. Mice with total absence of 24,25(OH)2D have a high risk of early neonatal death related to 1,25(OH)2D-mediated hypercalcemia and may display transiently impaired bone mineralization [100]. Recent data also suggest that the healing process of fractures is delayed in these mice [105].

The clinical implications are clear as there is universal consensus that all infants and most children need about 400 IU (or 600 IU for older children) of vitamin D per day to prevent rickets [1]. The vitamin D requirements of adolescents, pregnant women and adults are less well defined and the topic is still under debate. For the prevention of falls and fractures, there is greater consensus about the efficacy of daily 800 IU of vitamin D together with adequate calcium intake for institutionalized and vitamin D deficient elderly subjects [106]. Whether this also applies for otherwise healthy postmenopausal women or still mobile elderly subjects is unclear [107].

**Consensus Statements:**

1. Vitamin D is essential to prevent rickets and osteomalacia.
2. Vitamin D metabolites other than the active metabolite might have a role in fracture repair.
3. Vitamin D supplementation with adequate calcium intake can decrease the incidence of fractures in elderly, vitamin D deficient subjects.
4. Elimination of nutritional rickets remains a high public health priority.

**Research agenda**

1. Complete our understanding of the mechanisms of action of vitamin D in the intestine, with specific reference to its interaction with factors such as Fibroblast Growth Factor 23 (FGF23), vitamin K, and the intestinal microbiota.
2. Determine optimal values for vitamin D at all ages and conditions for skeletal health.
3. Develop a better understanding of the interaction between vitamin D status, e.g. serum 25(OH)D, and calcium intake in the development of nutritional rickets along with other possible markers of vitamin D status and other aspects of nutrition.

### Vitamin D as a risk factor for non-skeletal health

#### Cancer

One of the more intriguing aspects of the pleiotropic effects of vitamin D is its putative relationship to cancer [108, 109].

Preclinical cell and animal studies provide a compelling story and a strong rationale for a benefit of vitamin D and avoidance of vitamin D deficiency for cancer risk and outcome [108, 109]. Human observational and association studies are mixed regarding the benefits of vitamin D, but avoidance of vitamin D deficiency seems to more clearly indicate a health benefit [108, 109]. Several RCTs were discussed at the meeting especially the ViDA trial [110], but the results of other RCTs were not all available until they were published after the meeting. The trials generally concluded that risk of developing cancer was not statistically reduced by vitamin D supplements [110–112]. However, potential weaknesses in these studies included the fact that the vitamin D dose may not have been high enough and especially the time of follow-up was not long enough to see a reduction in the risk of developing cancer. It is important to emphasize that the control group receiving a placebo was not vitamin D deficient; thus studies tended to compare subjects with adequate circulating vitamin D concentration to subjects treated with supplements that achieved higher levels of circulating 25(OH)D.

In the recently published meta-analysis of the effect of vitamin D on incidence and survival of cancer patients, data also showed no significant reduction in cancer incidence [112]. However, vitamin D supplementation in highly significant findings reduced total cancer mortality. For total cancer mortality, five trials were included in the analysis with 1591 deaths; 3-10 years of follow-up; and 54-135 nmol/L of attained levels of circulating 25(OH)D in the intervention group. The summary RR was 0.87 (95% CI, 0.79-0.96; P = 0.005; I2 = 0%), which was largely attributable to interventions with daily dosing (as opposed to infrequent bolus dosing).

In a recent nested case-cohort study from Japan, a lower overall risk of cancer was found in men and women with higher levels of 25(OH)D [113]. Many previous studies have investigated the association of vitamin D with specific tumors. For example, cancers of the liver have been found to be associated with low 25(OH)D levels in cohort studies. In some studies, no association of vitamin D was found with prostate cancer, but there was a strong association between low vitamin D levels and high-grade prostate cancers [114].

CYP27B1, the final activating enzyme in the synthesis of calcitriol, is not only present in the kidney, but also expressed in non-renal sites as is the VDR. These non-renal sites include tumor cells themselves as well as cells in the tumor microenvironment [115].

Inhibition or mutations that inactivate the CYP24A1 enzyme lead to excessive 1,25(OH)2D production and hypercalcemia. Use of CYP24 inhibitors such as ketoconazole, liarazole and genistein among others have been shown to increase the anti-cancer activity of vitamin D in cell and animal models [116]. A recent study used a designed small molecule inhibitor of CYP24A1 known as CTA091 [117]. To avoid the systemic effects, a tumor targeted nanoparticle delivery system was developed to treat epidermal growth factor receptor-tyrosine kinase inhibitor (EGFR-TKI) lung cancer resistant to erlotinib. Delivery of vitamin D based drug payloads with CYP24A1 inhibitors via a tumor-targeted system was effective without causing side-effects and appears to be promising as a new therapy for EGFR TKI resistant lung cancer.

The goals of endocrine therapy of estrogen receptor positive (ER+) breast cancer are to deprive the cancer of the driving force for growth, namely estrogens. Three clinical approaches include: 1) using aromatase inhibitors (AIs) to prevent estrogen synthesis; 2) using selective estrogen receptor modulators (SERMs) to block estrogen binding to the ER; and using down-regulators of the ER (SERDs) to decrease the tumor concentration of ERs. Calcitriol has been shown to mediate all 3 activities [118]. Calcitriol or dietary vitamin D rapidly and equivalently inhibited the growth of mouse and human breast cancer in various mouse models [119, 120].

Vitamin D or its analogues demonstrate cell autonomous activity directly by acting via the VDR in tumor cells. Vitamin D can also regulate tumor cell behavior indirectly by acting on various cells in the microenvironment including stromal cells, adipose tissue and inflammatory cells, etc. Actions on the breast micro­environment contribute to vitamin D effects to inhibit breast cancer cell growth [121, 122]. In studies of colon cancer [123] and pancreatic cancer [124], an important additional anti-cancer activity of vitamin D via unique effects on cells in the micro-environment was demonstrated.

In experiments with the non-metastatic breast cancer cell line FARN168, studies showed that knocking down the VDR allowed the cell to transition into a metastatic cell [125]. Further evaluation demonstrated that restoring the VDR to the metastatic cells (VDR rescue) returned the cells to the non-metastatic state. Overexpression of the ID-1 gene, a gene commonly overexpressed in cancer cells, was identified as overexpressed in the VDR knockdown cells and reduced in the tumor cells when VDR expression was rescued suggesting that regulation of ID-1 by vitamin D contributes to its action to improve survival by inhibiting metastasis.

In a small trial including 29 cancer patients in which subjects received either high or low dose (placebo) vitamin D supplements, vitamin D supplementation was able to decrease circulating levels of 27-hydroxycholesterol, a recently identified endogenous SERM that can stimulate proliferation of breast cancer cells [126]. This study hypothesized yet another anticancer mechanism of vitamin D, acting by inhibition of the synthesis of a breast stimulatory endogenous SERM, likely by CYP27A1 inhibition.

A role of vitamin D has also been observed in colorectal cancer [127]. To identify optimal concentrations for colorectal cancer risk reduction, the association between circulating 25(OH)D and subsequent colorectal cancer incidence was studied in 17 prospective cohorts participating in the international Circulating Biomarkers and Breast and Colorectal Cancer Consortium [127]. Pooled data were correlated with 25(OH)D measurements obtained prior to cancer diagnosis. These findings demonstrated a strong, statistically significant, and robust inverse association between pre-diagnosis circulating vitamin D and colorectal cancer risk that was taken to substantially strengthen the evidence, previously considered inconclusive, for a causal relationship between circulating 25(OH)D and colon cancer risk. The study suggests that optimal circulating 25(OH)D concentrations for colorectal cancer risk reduction are 75-100 nmol/L, values somewhat higher than current Institute of Medicine in USA (IOM) recommendations for bone health.

**Consensus Statements:**

1. The relationship between vitamin D status and cancer is based on plausible mechanistic *in vitro* data, animal data and association studies in humans [128], especially for colon cancer where moderate effects of supplementation have been observed [123].
2. Published RCTs indicate that vitamin D supplementation did not significantly reduce cancer risk but did significantly improve cancer survival. However, weaknesses in the trial designs provide a cautionary note.
3. Appropriate selection of subjects (perhaps starting with a high-risk population) and other variables should be considered as components of optimal design.
4. Studies to determine the effect of vitamin D on cancer risk should be conducted for longer than 3-5 years, given the time course of oncogenesis.

**Research agenda**

1. Based upon observational data and the results of ongoing studies, additional RCTs are needed. These RCTS should take advantage of lessons learned from ongoing studies that are probably not optimally designed.
2. Long-term studies (5-10 years) are needed to assess whether vitamin D supplementation may prevent cancer.
3. Studies are needed to determine whether higher levels of 25(OH)D lead to improved survival among patients with cancer.

#### Celiac disease

Celiac disease (CD) is an autoimmune disorder of the gastrointestinal tract due to an immune response against gluten-containing grains in genetically susceptible individuals [129] with genotypes encoding the Human Leukocyte Antigen (HLA) class II molecules HLA-DQ2 and HLA-DQ8 [130]. The two markers of CD are a reduction in villous surface area of the small intestine and the appearance of anti-transglutaminase-2 antibodies in the plasma. Intercellular tight junctions are also altered in CD [131–133].

Low vitamin D levels have been linked to aberrant immune function in gastrointestinal diseases, including CD, inflammatory bowel disease (IBD) and dysbiosis [134, 135]. The vitamin D receptor (VDR) is highly expressed in the intestine. The activated VDR regulates innate immune response, promotes immune tolerance in the gut and also plays a role in maintaining intestinal barrier function, through regulation of the expression of the tight junction proteins. Vitamin D also regulates the gut microbiota, by controlling the composition of the gastrointestinal microflora [136, 137]. Specifically, data from animal models suggest that in the absence of the VDR or the ability to produce 1,25(OH)2D, unregulated inflammation of the gut results in an environment that supports the expansion of noxious bacteria in the Proteobacteria phylum, including the Helicobacteraceae family members, that out-compete beneficial members of the Firmicutes and Deferribacteres phyla.

**Consensus Statements:**

1. Vitamin D might be involved in the pathogenesis of CD in line with its potential role in the immune response, as it occurs in other autoimmune diseases.
2. Due to the heterogeneity in study design, different serum baseline levels of vitamin D and different doses of vitamin D administered in studies in these settings, harmonization of these variables is prerequisite to interpretation of the potential role of vitamin D in autoimmune diseases.

**Research agenda**

1. To determine whether vitamin D status influences/prevents CD in susceptible individuals.
2. To design intervention studies on potential benefits of vitamin D supplementation in CD.

#### Diabetes mellitus

The prevalence of diabetes mellitus among patients treated for osteoporosis varies widely, from less than 10 % in Europe to more than 25 % in some US populations [138].

Increased fracture risk was observed in both type 1 (T1DM) or type 2 (T2DM) diabetes. The rate of hip fracture risk is up to 7-fold higher in patients with T1DM and about 1.3-fold higher in patients with T2DM. It is probably due to detrimental effects of impaired glucose metabolism on bone health as well as to an increased risk of falls or other traumatic events, frequently reported in diabetic patients [139, 140].

Changes in mineral bone density (BMD) are not similar in T1DM and T2DM patients, and often conflicting. In the Melbourne Collaborative Cohort Study, vitamin D status was inversely associated with the risk of T2DM, and this association did not appear to be explained by reverse causality [141].

A potential role for abnormal vitamin D status in changes of glucose homeostasis has been described [142]. It has been demonstrated that vitamin D deficiency is detrimental to the synthesis and secretion of insulin in animal and human studies. In several, but not all, human observational trials, an inverse correlation was seen between vitamin D with insulin insensitivity, prediabetic states and dysglycemia. Recently, data from non-interventional observational studies have shown a negative relationship between the vitamin D status and parameters of insulin insensitivity and incidence of T2DM [143]. In a meta-analysis of 21 studies, the association between vitamin D and parameters of insulin insensitivity and incidence of T2DM were demonstrated [144].

From a pathophysiological point of view, vitamin D levels and thioredoxin interacting protein were associated with different beta-cell dysfunction markers, indicating their potential abilities to predict the beta-cell status in people with diabetes [145].

Evidence from observational studies as well as recent clinical trials support the beneficial effect of vitamin D on glycemic control and subsiding systemic/vascular inflammation in T2DM [146, 147]. Moreover it was showed that diabetic subjects might respond differently to vitamin D supplementation according to their VDR Fokl genotypes [148].

Vitamin D supplementation is beneficial for the reduction of hs-CRP in T2DM subjects but does not have a significant influence on Tumor Necrosis Factor α (TNF-α) and interleukin – 6 (IL-6) in T2DM subjects [149].

With regard to the effect of vitamin D supplementation on fasting plasma glucose (FPG), insulin resistance and prevention of T2DM in non-diabetics, no significant effect was observed on controlling FPG level, improving insulin resistance or preventing T2DM in non-diabetic subjects in a pooled meta-analysis [150]. On the other hand, vitamin D administration and improved vitamin D status was shown to improve glycemic measures and insulin sensitivity.

#### Obesity

Vitamin D deficiency is highly prevalent, especially in elderly obese individuals [48], with proven detrimental effects on bone and muscle health. Considering the inverse correlation between BMI and vitamin D levels and the large prevalence of obesity worldwide, vitamin D deficiency is a growing health concern in the obese, regardless of age group [151]. Adipose tissue is a direct target of vitamin D, which plays a role in modulating adipose tissue distribution and activity [152]. This is further confirmed by evidence of vitamin D receptors (VDR) in pre -adipocytes, adipocytes, in both subcutaneous and visceral adipose tissue [153]. Despite several epidemiological studies showing the existence of a close relationship between obesity and hypovitaminosis D, the mechanisms underlying this association are still largely unknown. Interestingly, it has been suggested that vitamin D may provide a protective effect in obese individuals, by reducing systemic inflammation. Therefore, vitamin D may be considered a protector for obesity and related clinical conditions. However, only a few and mostly underpowered randomized clinical trials have been conducted to test the effectiveness of vitamin D supplementation in facilitating weight loss or other metabolic outcomes in obese people. Similarly, recent evidence linking vitamin D deficiency to non-alcoholic fatty liver diease (NAFLD) progression has led to the hypothesis that vitamin D may play a protective effect, controlling hepatic inflammation, with decreased liver mRNA expression of resistin, IL-6 and TNF-a [154]. However, also in this case, clinical trials have yielded inconsistent results.

A recent meta-analysis of 35 studies (including 17,245 persons) showed that serum vitamin D is inversely associated with body fat mass (FM), but the analysis of RCTs does not support the hypothesis that vitamin D supplementation augments body-fat loss [155].

Bariatric surgery has been proven to be the most effective treatment of morbid obesity, leading not only to a long-term weight reduction but also to a significant improvement of health-related quality of life and a reduction of overall mortality.

Biliopancreatic diversion (BPD) procedure causes the most severe and persistent reduction in serum 25(OH)D. A retrospective study revealed that 25(OH)D levels decrease over time with BPD even 9 years after surgery [156]. Therefore, prevention and treatment of hypovitaminosis D in patients undergoing bariatric surgery is crucial in order to prevent bone loss and other complications. Regardless of bariatric surgery procedure choice, all patients need periodic assessment of their vitamin D status.

**Consensus Statements:**

1. Both obesity and diabetes are known to be associated with vitamin D deficiency, but the mechanisms involved have not yet been clearly elucidated.
2. Supplementation of vitamin D has not been shown to improve outcome measures of diabetes and obesity.
3. Bariatric surgery has several key outcomes related to vitamin D metabolism, including reduced absorption.
4. Severe vitamin D deficiency is frequent in patients after bariatric surgery and correction requires much higher doses compared with obese people, which in turn requires larger doses than non-obese individuals.
5. Calcium malabsorption may persist after correction of vitamin D deficiency because of reduced active intestinal calcium absorption.
6. These patients have an increased rate of fracture compared with obese patients of the same age.

**Research agenda**

1. Identify the molecular mechanisms associated with lower circulating 25(OH)D levels in obesity.
2. Investigate whether there are optimal thresholds to prevent or improve the onset and outcome of T1DM and T2DM.

#### Multiple sclerosis

Multiple sclerosis (MS) is an autoimmune disease that alters the central nervous system, leading to the presence of focal areas of inflammation and demyelination [157]. Increasing evidence suggests that several environmental factors including inadequate vitamin D levels are associated with the progression of MS [158]. Individuals with adequate levels of vitamin D appear to have reduced prevalence, activity and progression of MS [159]. Several observational studies have explored factors affecting vitamin D levels (e.g. sunlight exposure, latitude and diet) and support an association between elevated vitamin D levels and reduced MS disease severity [160, 161]. In experimental studies examining the effect of vitamin D supplementation, it has been observed that low serum vitamin D levels can exacerbate MS symptoms and are associated with higher relapse rates, new lesions and greater disability [162–165]. Furthermore, daily cholecalciferol supplementation has recently been shown to improve depressive symptoms in patients with relapsing remitting MS [166]. Lower vitamin D levels were associated with higher depressive scores and vitamin D supplementation improved these symptoms [166].

The majority of studies in the MS setting suffer from substantial heterogeneity in terms of study design, baseline serum vitamin D levels and outcome measures, making cross-comparison difficult and results inconclusive. To address these shortcomings, systematic reviews have been undertaken to examine the effect of vitamin D supplementation on MS; however, some of these have been limited by lack of a range of vitamin D status and not accounting for bias [167] or failure to assess cytokine outcomes or baseline vitamin D on outcomes [168]. A third systematic review has attempted to address some of these former weaknesses [169]. In this meta-analysis which included 10 studies, vitamin D supplementation was compared to placebo or low dose vitamin D. Overall, disease measures improved to a greater extent in patients with lower baseline serum 25(OH)D levels. In 3 out of the 10 studies included, an improvement in disease measures was more apparent in patients with lower baseline vitamin D levels.

A number of Mendelian randomization studies identified a link between genetically low vitamin D status and MS (n=95) and another autoimmune disease, T1DM (n=1). This makes a causal link between vitamin D status and these diseases likely, particularly for MS.

#### Pregnancy and lactation

Mounting evidence places vitamin D as a central and necessary nutrient for conception, normal placental function, and maternal and fetal immune homeostasis. It is also a key factor for continued well-being during lactation [170, 171]. Outcome data from 4 randomized controlled trials during pregnancy and lactation [172, 173] as well as other studies [174, 175], collectively support the Barker hypothesis [176, 177] that identifies vitamin D as a vital factor in maternal and infant well-being.

Alluding to general findings that skin color is important in vitamin D homeostasis, women most affected by suboptimal vitamin D status are those of darker skin pigmentation or with limited sunlight exposure. In the US, for example, African American women have the most significant vitamin D deficiency and the worse pregnancy outcomes. While many other factors could account for worse pregnancy outcomes among African American women, the potential negative impact of vitamin D deficiency looms large as a contributing factor [178]. After pregnancy, the need for adequate vitamin D in mother's milk ensures not only adequate vitamin D delivery to the breastfeeding infant, but it also ensures that immune competence aided by vitamin D is optimized through mother's milk [170, 179]. Ensuring adequate vitamin D nutrition during pregnancy and lactation should be an integral component of global public health policies.

**Consensus Statements:**

1. Vitamin D adequacy for mothers and infants is important.
2. The implication of vitamin D deficiency during pregnancy is not fully understood. Results from meta-analyses suggest that improving maternal vitamin D status can decrease pregnancy outcome risk and may have long-term beneficial effects in the offspring, such as asthma.

**Research agenda**

Define the optimal vitamin D status before conception, during pregnancy and lactation for optimal health of the mother and her offspring.

#### Degenerative neurological diseases

Accumulating evidence indicates that vitamin D hormone may play a role in aging and age-related cognitive decline [180, 181]. Human studies have noted a positive association between vitamin D levels and cognitive functions in aged individuals. Annweiler and colleagues reported that supplementing elderly individuals with vitamin D during 16 months improves executive function [182, 183]. Several neurological disorders have been possibly connected with deficient vitamin D status, including multiple sclerosis [184], Parkinson's disease [185], and Alzheimer’s disease (AD) [186].

In animals, vitamin D deficiency during the early stages of life has a strong influence on brain function when maturity is reached, suggesting that vitamin D deficiency, at different time-points and for varying periods of time, could be a component of neurodegenerative diseases such as Alzheimer's disease [180, 187]. In addition, in vitro and in vivo experiments have demonstrated the following about vitamin D neurophysiology: 1) upregulation of the expression of several neurotrophins; 2) stimulation of the secretion of anti- inflammatory cytokines; 3) modulation of neurotransmitters such as acetylcholine, dopamine and serotonin; 4) regulation of growth factors such as glial cell-derived neurotrophic factor and neurotrophin nerve growth factor. It has also been observed that vitamin D is involved in 1) synaptogenesis, 2) axonal growth, 3) intraneuronal calcium homeostasis, and 4) inhibition of nitric oxide synthase [188].

Animal models of AD and in vitro studies have demonstrated that vitamin D 1) enhances cerebral clearance of human amyloid peptide from mouse brain across the blood -brain barrier and 2) prevents amyloid- induced alterations in cortical neurons [189]. Vitamin D3 deficiency increases spatial learning deficits in these animal models. Conversely, vitamin D3 supplementation decreases pathological markers of the disease, such as amyloid deposition, but also alters the expression of various genes, some of which are involved in the regulation of estrogen and insulin-like growth factor 1 (IGF-1) production [190, 191].

Hippocampus-dependent learning and memory formation are associated with increased cell proliferation and neurogenesis. A reduction of hippocampal neurogenesis has been shown to be involved in the pathogenesis of AD [192]. In this regard, vitamin D appears to improve working memory and endogenous neurogenesis, but only when presented before the onset of the major symptoms. Vitamin D deficiency, on the other hand, increases the amyloid burden and impairs neurogenesis in the brains of male transgenic animals [193]. Thus, these studies suggest critical windows of time, hormonal interactions, and gender-specific effects of the processes in which vitamin D may be important [190, 191]. This conclusion is supported by additional studies between male and female animals with AD [190, 191, 193]. In these studies, vitamin D supplementation was observed to induce a better cognitive performance in late stages of female animals and in the early stages of males, but not in the late stages of male animals [190, 191, 193]. The exact molecular mechanisms by which vitamin D could be beneficial in AD are not yet well understood but collectively, the results suggest some role [193, 194].

**Consensus Statement:**

1. Chronic deficiency of vitamin D in animals could predispose to the development of chronic degenerative neurological diseases.
2. Chronic degenerative neurological diseases in humans have been associated with vitamin D deficiency but a potential causative role continues to be elusive.

**Research agenda**

Pre-clinical and clinical studies are warranted to understand the role of vitamin D in neurological diseases.

#### Falls

Falls are complex events that often involve multiple deficits. Of the 10 leading causes of falls, the strongest predictors, in descending order are: 1) muscle weakness, 2) history of falls, 3) gait deficit, and 4) balance deficit [195]. Vitamin D deficiency mimics several of the age-related changes that occur in muscle and that are associated with increased risk of falling. In vitamin D deficiency, there is a preferential loss of type 2 muscle fibers [196], fewer intramuscular vitamin D receptors [196], and reduced balance [197]. Vitamin D has been shown to be associated with the prevention of a series of complications of chronic diseases, such as fracture and fall risks [198]. A recent meta-analysis of 29 randomized placebo-controlled intervention trials revealed that vitamin D supplementation improved muscle strength [199]. Vitamin D supplementation did not increase muscle mass or power but this evidence was limited to the few available studies that included these measurements [199]. Vitamin D supplementation has also improved balance in older adults, as measured by reduced sway [197].

The evidence so far evaluating the effect of vitamin D supplementation on reducing the risk of falls is mixed. A 2011 meta-analysis examining the role of vitamin D dose concluded that a dose of 700 to 800 IU was required to lower risk of falls and that with optimal dosing, the risk reduction was on the order of 15% [200]. Several trials then tested 800 IU per day. In one conducted in postmenopausal women, neither 800 IU per day (nor 50,000 IU twice monthly, also tested) influenced the risk of falling [201]. A similar negative result was observed in another 800 IU trial in over 400 women aged 70 to 80 years [202]. However, an intervention study testing multiple vitamin D supplement doses ranging from 400 to 4,800 IU per day found significantly fewer falls among postmenopausal women taking doses of 1,600 - 3,200 IU per day [203]. A limitation of this study was its relatively small sample size.

The ViDA trial that tested the effect 100,000 IU per month on fall risk in 5,108 adults over a 3.3-year period did not observe a significant effect. Outcomes of trials testing even higher, less frequent doses of vitamin D will be discussed in a different section. A factor that may affect efficacy of supplementation is the vitamin D status of the study population. The trials cited above involved participants who were vitamin D replete or only mildly insufficient [201, 202]. An exception to this was a trial conducted in Brazilian women who had a low mean serum 25(OH)D level of 37.4 nmol/L [204]. In these women, supplementation with 1,000 IU of vitamin D3 per day over a 9-month period increased the mean serum 25(OH)D level to 27.5 ng/ml and reduced risk of falling by nearly a half. A meta-analysis of vitamin D and falls trials concurred that trials in populations with low 25(OH)D levels showed a positive effect of supplementation whereas those in replete populations were null [205]. Additional trials in adults with low 25(OH)D levels are needed to confirm this finding.

**Consensus Statements:**

1. Vitamin D deficiency is associated with muscle dysfunction and falls in the elderly.
2. Vitamin D supplementation can reduce or increase the risk of falls in individuals who are moderately deficient in vitamin D.
3. The ability to ‘see’ or ‘not see’ an effect on falls and perhaps other outcomes of vitamin D actions may depend upon whether the population studied is or is not vitamin D deficient. As a threshold nutrient, it would not be expected to show beneficial outcomes unless the population studied is deficient in vitamin D.

## Therapeutics

### Dichotomy between observational studies and randomized trials in establishing causality

In assessing causality in human diseases, at the top of the Hierarchy of Scientific Evidence pyramid sits meta-analyses of well conducted Randomized Controlled Trials (RCTs), followed by RCTs. Below RCTs sits Mendelian randomization trials, long-term large observational studies i.e. cohort and case-control studies. The lowest evidence comprises cross-sectional studies, case series and case reports [206]. Unfortunately, this pyramid has led to a false dichotomy that in assessing causality one must choose between RCTs and observational epidemiology.

The primary focus of vitamin D research is to test the hypothesis that poor vitamin D status, as measured principally by serum total 25(OH)D concentration, increases the risk of developing a disease or condition. However, is the serum total 25(OH)D concentration the appropriate measure of risk in all diseases thought to potentially be causally related to vitamin D status? It is certainly true for rickets, but is it true for non-skeletal diseases? The lack of answers to these questions is fundamental to controversies in the vitamin D field, which is in part due to the lack of standardized 25(OH)D measurements in vitamin D research, affecting both epidemiological studies and RCTs.

A critical question: if the currently ongoing large vitamin D RCTs fail, does that mean that poor vitamin D status is *not* related to a particular disease of interest? Clinical trials can fail for many reasons, including the study design. It may be that risk was inappropriately measured and/or defined in the study population, so that those included were not at high risk. This aspect of study design is particularly important in nutrient research, a point the late Prof. Heaney made so well [207, 208].

RCTs are essential in testing various components of the various hypotheses related to disease outcomes and measures of vitamin D status in humans and in animal models (e. g. does poor vitamin D status, or an abnormality of vitamin D metabolism lead to an increased rate of atherosclerosis in monkeys on a high fat diet?). In this regard, some hypotheses that arise may only be answered by epidemiological or *in vitro* studies. As a result, it may be necessary to develop a chain of causation with data from many different sources. Just such a chain of causation was needed to conclude that a diet high in saturated fatty acids and cholesterol leads to increased serum total cholesterol levels and, finally, to an increased risk of Coronary Heart Disease (CHD) [209].

Systematic review methodology is an important process in research synthesis. However, it is important to conduct large RCTs in nutrition, including vitamin D, when we adequately know how to measure vitamin D status and define risk of a study population. There is a very real risk that if recently published or currently ongoing large RCTs fail, the entire field may lose substantial credibility not only with the public, but also with grant funding agencies. In this regard, we need to return to a balanced approach to vitamin D research that is based on establishing a chain of causation based on data from all possible sources and which emphasizes assay standardization.

#### Value of observational/association studies

For any area of medical research, observational and association studies often provide the first evidence that a subject needs to be investigated in depth. There are several types of studies in this category: ecological, cross-sectional, case-control, and cohort studies [210]. The latter can have either an extended longitudinal study design. Ecological and cross-sectional studies often inform cohort studies and RCTs. They may be large, and if consistent across many varying populations can provide important information. In addition, they may provide essential guidance in designing more definitive studies. Association studies are often able to include much larger populations than RCTs. They can also be analyzed by meta-analysis to provide hypotheses for RCTs. As in all other areas of medical research, careful review of observational and association studies will improve RCT design.

Many observational and association studies of vitamin D have led to hypotheses that vitamin D exerts effects beyond bone and mineral metabolism. For example, low serum concentrations of 25(OH)D were found to be associated with a higher incidence of cardiovascular disease [211, 212], in addition to the known associations of serum lipids, smoking, diabetes mellitus, obesity, and high blood pressure [213]. Several plausible potential mechanisms have also been identified in laboratory studies. Association studies help in RCT design by determining which other potential independent variables that must be accounted for in the trial.

#### Value of randomized controlled trials

A randomized controlled clinical trial is defined as a prospective study comparing the effect (i.e. benefit-to-risk profile) of one intervention against another (either control or active), randomly assigned in humans [214]. Meta-analyses of well performed RCTs are at the top of the hierarchy of research evidence to advance clinical care. RCTs share many components with other study designs (e.g. need for an underlying hypothesis, selection of a population of interest, comparison of well-defined interventions, definition of outcomes) but, when well designed, they differ from other study designs in a single important element: a low probability for bias.

When "comparing" interventions, clinical trials should respect the principle of equipoise, which is a defined as a true state of uncertainty among the expert medical community and the researchers regarding the comparative efficacy-safety balance of each arm in a trial. Although equipoise is an elegant principle, it is difficult to implement in practice for a number of reasons, the main one being persistent researcher bias [215].

A distinct advantage of vitamin D trials, unlike other interventions with nutrients, is that serum 25(OH)D concentration can be measured to assess the effect of and adherence to the intervention (vitamin D supplementation). Importantly, the serum 25(OH)D concentration in RCTs is primarily the direct result of the study intervention and not a marker of health or disease, as in the observational studies with vitamin D. Therefore, the trial result (whether positive or negative) associated with a higher vs lower serum 25(OH)D concentration can be attributed to the trial intervention, emphasizing the importance of standardized measurements of 25(OH)D in RCTs.

The main limitation of a clinical trial is that it is designed, powered and conducted for a single primary outcome in a narrow population to test a very specific intervention. For example, in a recent study, monthly vitamin D3 supplementation of 100,000 IU for a median of 3.3 years did not prevent cancer [216]. That result does not necessarily mean that vitamin D3 supplementation over 5 or 10 years will not prevent cancer, as it may take longer for cancer to either develop and progress to a clinically apparent state.

A properly planned and executed RCT is a powerful experiment for assessing the effect-to-safety balance of an intervention and to guide clinical care. On the other hand, poorly designed, conducted, analyzed and reported trials can be misleading, potentially more than other study designs. Therefore, a successful trial is not one that only achieves a positive result, but one that reliably answers the primary question the trial was designed and conducted to answer. Finally, without supportive evidence, no single RCT (even a well conducted one) ought to be considered definitive and consistency among RCTs should be sought. Researchers and clinicians also need to balance RCT results with results from laboratory, animal and observational studies to establish a chain of causation.

#### How to design an ideal randomized controlled trial with vitamin D

Several factors can explain a lack of vitamin D efficacy on clinical outcomes in recent RCTs. Low doses as well as high doses may be inefficient, with a U-shape relationship existing between vitamin D doses and outcomes, for instance, fall risk or mortality [203]. A feature of vitamin D overdosing is the increased risk of fracture and falls observed with the yearly or monthly administration of large doses of vitamin D (corresponding however to a mean daily dose of 1,350 IU or 2,000 IU, respectively) [217]. These results provide important information for future clinical trial design. Additional secondary and exploratory outcomes should also be considered to further identify other potential effects of vitamin D.

The population included in the study is of major importance. A vitamin supplementation is more likely to produce a clinically significant effect in patients with hypovitaminosis D than in community dwelling healthy vitamin D sufficient individuals. Thus, vitamin D supplementation should be tested in vitamin D deficient subjects.

For fracture risk reduction, most positive trials have used a combination of calcium and vitamin D, emphasizing the importance of cofactors in vitamin D efficacy. The design of an ideal RCT with vitamin D should be adapted to the targeted disease and to the selected primary outcome. It could include a non-intermittent regimen with one or 2 moderate doses. The duration should be sufficient to record an adequate number of events, but limited to ensure optimal adherence [218]. Ideally, the population should be, or predicted to be, deficient in vitamin D. Controls may be either placebo or poorly effective low doses of vitamin D.

The number of subjects should be large enough so that the 95% confidence intervals are smaller than the expected effects. This implies also a full control of confounding factors and co-morbidities. Taken together, these various conditions suggest that an ideal RCT with vitamin D will be difficult in terms of patient recruitment, and expensive with, in addition, the risk of enrolling a population that is too selective. The more highly selective the population is, the greater uncertainly as to its general applicability. However, selection of a population expected to be moderately to severely deficient (serum 25(OH)D <30 nmol/L) would allow multiple outcomes to be studied in an RCT of vitamin D supplementation.

#### Assessing risk of bias in vitamin D RCTs

The VDR is found in essentially all cells of the body. The VDR ligand regulates the transcriptional activity of thousands of genes. Cellular and animal studies have documented the major impact vitamin D has through the active ligand 1,25(OH)2D and receptor VDR have on numerous biochemical and physiologic pathways and processes. Association studies in humans suggest that low serum 25(OH)D concentrations contribute to a large and growing list of medical conditions. However, RCTs testing the ability of vitamin D to exert beneficial effects on these medical conditions, other than the treatment of rickets/osteomalacia, have been remarkably unimpressive overall. Why? There are at least two possible explanations. The first is that the impressive data from cells and animals do not translate to the human condition. One might relegate this possibility to highly unlikely. A second explanation is that flaws in the design, conduct, analysis, and reporting of the RCT can bias the results achieved.

In 2005, the Cochrane Collaboration began a process to develop a new tool for assessing risk of bias in RCTs [219]. This tool has been broadly adapted with minor modifications in meta-analyses of a variety of vitamin D supplementation (and other) RCTs. As tabulated by Higgins et al., there are 6 major bias domains [219]. The first is selection bias. This domain includes an assessment of the methods involved in randomization of participants. Moreover, the size of the trial needs to be large enough to minimize differences between the groups and the power to robustly answer the question being posed. Equally important is whether the subjects selected to participate in the trial are appropriate for the question being asked. Expecting a benefit of vitamin D supplementation in subjects with normal serum vitamin D concentrations is probably unrealistic. The second is performance bias. This focuses on the blinding of participants and personnel managing the study. A risk in vitamin D RCTs is if participants sense they are receiving a placebo they begin taking or altering their intake of vitamin D and calcium, minimizing their difference with those in the active arm of the study. Detection bias is the third domain, and includes the assessment of measures used to blind those personnel responsible for evaluating the outcome from knowledge of the intervention. That said, determining the serum 25(OH)D concentration before and after the intervention in a vitamin D RCT is critical for determining not only compliance, but whether a response to the intervention can reasonably be expected. However, such testing will dramatically increase the expense of an RCT and, therefore, has the potential of limiting study size and power. Attrition bias is the fourth domain and includes how incomplete data are handled. In a lengthy trial, drop outs and decreased compliance complicate the analysis. Should subjects assigned to vitamin D supplementation, but who stopped or limited their vitamin D doses be considered as part of the active arm of the study, even if their 25(OH)D levels do not increase? Reporting bias is the fifth domain. The risk here is that of selective reporting. A trial may not reach its primary endpoint, but with data mining, other results may be found to be significant. Can results from a vitamin D RCT for treatment of osteoporosis be used to determine whether there was a reduction in cancer incidence? Finally, there is a category listed as other biases. This could include sources of funding. For vitamin D RCTs, this could also include design issues such as duration of the trial, doses expected to be adequate to achieve the desired effect, and the appropriateness of the dosing intervals.

A moderate to high risk of bias confounds many of the RCTs that we rely on for determining the efficacy of vitamin D supplementation on a number of medical conditions that animal and human association studies suggest could benefit from such supplementation. The risk of these biases impacting the interpretation of the reported results is compounded by trials in which participants are: 1) not preselected for vitamin D deficiency; 2) adherence is poorly monitored and/or not adjusted for in the analysis; 3) the dose of the vitamin D provided is not sufficient to achieve a significant increase in serum 25(OH)D concentrations; 4) the dosing intervals are too infrequent to provide a steady level of serum 25(OH)D concentrations; and 5) the effect of the administered dose on 25OHD is not determined and/or adjusted to ascertain that an effective level of 25(OH)D has been achieved. The degree to which these concerns have resulted in the large number of negative or minimally effective results with vitamin D supplementation for extra-musculoskeletal conditions is unclear. Future investigation using stricter criteria for RCT design, conduct, analysis, and reporting are necessary to address these concerns.

#### Ideal biochemical target when replacing vitamin D

The ideal biochemical target for replacing vitamin D can be identified by interpolating recommended dietary allowances for vitamin D with evidence from pre-existing randomised controlled trials to balance concerns regarding efficacy and safety.

The IOM has set the recommended dietary allowances for vitamin D at 600 IU/day for adults aged 50-70 years and 800 IU/day for those aged >70 years, which would correspond to a serum 25(OH)D level of 50 nmol/L, sufficient to maintain bone health in 97.5% of North Americans [35, 48]. These are population-based recommendations, however, whereas data from meta­analyses of randomised controlled trials suggest that additional and optimal benefits may accrue at levels of 60-75 nmol/L and 90-100 nmol/L for musculoskeletal and non-musculoskeletal outcomes, respectively. For example, using a daily dose of vitamin D, falls are reduced at serum 25(OH)D of about 60 nmol/L, while non-vertebral fractures are reduced at levels 75 nmol/L. For multiple sclerosis, as an example of non­musculoskeletal outcomes, it seems a higher biochemical target of at least 100 nmol/L is needed for prevention. For a typical older adult, achieving a serum 25(OH)D level of 75 nmol/L requires a daily vitamin D dose of at least 800-1,000 IU. The rise in serum 25(OH)D is also slower in individuals with higher baseline intake.

### Results from recent RCTs

In the 2000s, emerging evidence from observational epidemiological studies of significant inverse associations between vitamin D status, as measured by serum 25(OH)D concentrations, and a range of non-skeletal diseases [220], combined with the null findings from the Women's Health Initiative that a low dose of vitamin D (400 IU/day) did not prevent colorectal cancer [221]. This led to the design and funding of several large-scale RCTs to determine if higher doses of vitamin D supplementation (2,000 IU/day or the monthly equivalent) prevented a variety of diseases including cardiovascular disease, cancer and mortality [222].

Several large vitamin D supplementation trials are either underway or have recently published their findings. The purpose of these trials is to investigate the effects on incidence of major chronic diseases of long-term supplementation with vitamin D doses that are much higher than currently recommended by most organizations. Although RCTs with "hard" endpoints (incidence of disease, death) are considered to provide the best evidence for causality, there are several factors that may affect the outcomes in the large vitamin D RCTs. Unlike with pharmaceutical agents, studies with nutrients such as vitamin D have specific issues that need to be considered when interpreting data from vitamin D RCTs.

#### VITAL Study

These considerations have been taken into account when planning currently ongoing or recently completed large population-based RCTs of vitamin D. In planning the Vitamin D and Omega-3 trial (VITAL) comprising 25,871 older men (aged >50 years) and women (aged >55 years), given a daily dose of 2,000 IU or placebo. The mean serum 25(OH)D concentration level at baseline was 77 nmol/L and 12.7% had levels below 50 nmol/L. In participants with repeat measurements after 1 year, mean serum 25(OH)D concentrations increased by 40 nmol/L at 1 year [223]. The primary study outcomes of cancer and major cardiovascular events were both negative after a median 5.3 years of follow-up. Secondary end-points for death from cancer, breast cancer, prostate cancer, colorectal cancer; the expanded composite end-point of major cardiovascular events plus coronary revascularization, myocardial infarction, stroke, and death from cardiovascular causes; death from any cause were also all negative. No excess risks of hypercalcemia or other adverse events occurred in the vitamin D group. However, the rate of death from cancer over time was significantly lower with vitamin D than with placebo (HR 0.79 [95% CI, 0.63 to 0.99], and HR 0.75 [95% CI, 0.59 to 0.96], respectively). In analyses restricted to deaths from cancer in patients with adjudication of the cause of death, HRs were 0.72 (95% CI, 0.52 to 1.00) over the total follow-up period, and 0.63 (95% CI, 0.43 to 0.92) when the first 2 years were excluded.

#### D-Health Trial

In the ongoing Australian D-Health trial, 21,315 older adults have been randomised to receive either 5 years of oral vitamin D (60,000 IU/month) or placebo and will be followed for a total of 10 years for total mortality, as a primary outcome, with multiple secondary outcomes including total cancer and colorectal cancer incidence, falls, fractures and infections [224].

#### ViDA Trial

The first of the vitamin D mega-trials to publish is the Vitamin D Assessment (ViDA) Study carried out in Auckland, New Zealand, 2010 to 2015. Like the VIDAL pilot trial in the UK [225] ViDA [226] has employed higher monthly doses of 100,000 IU compared with placebo (with an initial bolus of 200,000 IU in ViDA). The study enrolled 5,110 adults, aged 50-84 years, recruited mainly from family practices with vitamin D3 (2.5 mg or 100,000 IU) or placebo softgel oral capsules, mailed monthly to participants' homes. Outcomes were monitored through routinely collected health data and self-completed monthly questionnaires. The mean baseline serum 25(OH)D concentration was 66 nmol/L and the mean post-treatment serum 25(OH)D concentration obtained in ViDA was 116 nmol/L.

The results showed no beneficial effect of vitamin D supplementation on incidence of cardiovascular disease [226], falls [227], non-vertebral fractures [227] and all cancer [216]. However, beneficial effects from vitamin D supplementation were seen: for persistence with taking statins in participants on long-term statin therapy [228]; and also in bone mineral density [229], and arterial function (central blood pressure) [230], particularly in participants with low serum 25(OH)D concentrations. Beneficial effects were also seen on lung function (forced expiratory volume in 1 second) among ever smokers, and patients with Chronic Obstructive Pulmonary Disease (COPD) or asthma (especially if vitamin D deficient) [231]. The latter findings are consistent with several previous studies. The beneficial effect seen on statin persistence [228] is preliminary and needs to be confirmed by other studies.

The lack of effect could be due to the use of monthly, rather than daily or weekly supplementation, insufficient participants with vitamin D deficiency, lack of co-supplementary calcium or too short a follow-up period for chronic disease outcomes. However, the ViDA study did show that the beneficial effects for some outcomes such as BMD [229], FEV1 [231] and arterial function [230] are more pronounced in subjects with serum 25(OH)D concentrations of <50 nmol/L. These findings are consistent with several previous studies, including VITAL and D2d, which collectively suggest that vitamin D supplementation may only be beneficial in people who are deficient [232].

#### DO-HEALTH Trial

The European Vitamin D3 - Omega3 - Home Exercise - Healthy Ageing and Longevity Trial (DO-HEALTH) enrolled 2,157 older men and women aged >70 years and treated them with Vitamin D 2,000 IU per day, marine omega-3 fatty acid 1g/d and exercise in a 2x2x2 factorial design for 3 years with multiple study endpoints including functional and cognitive decline, falls and fractures [233].

#### FIND study

The Finnish FIND study randomised 2,495 older men and women to either 3,200 IU or 1,600 IU vitamin D per day for 5 years with cancer and cardiovascular disease as primary outcomes [234].

#### International Polycap Study 3 (TIPS-3)

Finally, the International Polycap Study 3 (TIPS-3) will enroll 5,000 older men and women at high risk of cardiovascular disease and randomize to 60,000 IU vitamin D per month versus a polycap containing thiazide, atenolol, ramipril, simvastatin or placebo in a 2x2x2 factorial design for 5 years with 5 years of follow-up, having major adverse cardiovascular events (MACE) as the primary endpoint [235].

#### Vitamin D and Type 2 Diabetes Study (D2d)

In a previous trial of vitamin D in vitamin D-deficient adults at risk of diabetes, a median daily dose of 4,000 IU (range 2,000 IU - 6,000 IU) was required to achieve a target serum 25(OH)D level of >75 nmol/L, because all trial participants were either overweight or obese [236]. In the ongoing Vitamin D and Type 2 Diabetes Study (D2d) in USA, in 2,423 individuals aged >25 years with risk factors for diabetes mellitus, a daily dose of 4,000 IU has been chosen with incident diabetes over 3 years being the primary outcome [237].

In conclusion, the equivalent daily doses in the large ongoing or recently completed vitamin DRCTs range from 2,000 - 4,000 IU and all aim for a target serum 25(OH)D concentration 90-100 nmol/L as non-musculoskeletal outcomes are primary study outcomes.

### Limitations of large vitamin D RCTs

1. A continuing problem with interpreting the data from large RCTs remains the lack of vitamin D assay standardized research data. Without such data, it will continue to be difficult to derive information about disease prevention in pooled subsets of patients from these large RCTs with mild or moderately severe vitamin D deficiency.
2. Many observational studies have found that the association of serum 25(OH)D concentration with some outcomes, such as cardiovascular disease and mortality risk, is not linear. Although the threshold level, after which little or no further reduction in risk is observed, has varied between studies (possibly due to the unstandardized 25(OH)D measurement methods), little additional benefit has been observed with concentrations higher than about 75 nmol/L [232]. It is well known that volunteers who participate in clinical trials are in general healthier and more health-conscious than the average population. Thus, in many RCTs a majority of the study population may already have sufficient vitamin D exposure at the study baseline from a healthier diet, from a more regular use of supplements or from more frequent outdoor activities. This was also observed in the CAPS study, where the starting level of 25(OH)D was 83 nmol/L and only 9.6% had concentrations <50 nmol/L (<20 ng/ml) [128]. In ViDA, the baseline 25(OH)D level was about 66 nmol/L (26.5 ng/mL) and 24.9% had insufficient levels (<50 nmol/L) [226]. In VITAL, the mean serum 25(OH)D concentration level at baseline was 77 nmol/L, and only 12.7% had insufficiency. However, none of the large trials have pre-screened the participants for low 25(OH)D concentrations.
3. If vitamin D is more accessible than 25(OH)D for internalization into cells, a stable concentration of vitamin D in circulation would be preferred for the optimal benefits of vitamin D [222]. Many of the large trials use an intermittent, monthly bolus dosing of vitamin D. Although an intermittent dosing is helpful for increasing compliance in taking the supplement, the wide fluctuations in circulating vitamin D do not reflect the more constant physiological exposures to vitamin D and may thus result in misleading findings from the trials using this supplementation regime.
4. Unlike in studies with pharmaceutical agents, in trials with dietary supplements the participants in the control group are exposed to the study agent to a certain extent. In the case of vitamin D, the exposure can come from the UVB exposure, from diet or from supplements that are also allowed in the placebo group due to ethical reasons. Fortification of foods with vitamin D and the more widespread use of vitamin D supplements can increase the average vitamin D exposure in populations. Although the exposure to vitamin D from other sources than study supplements may not differ between the experimental and control groups, the increased exposure can lower the proportion of those with low serum 25(OH)D concentrations in the control group and thus decrease the chances for finding beneficial effects. For example, in the CAPS study, those in the placebo group used higher doses of their own vitamin D supplements during the study than those in the vitamin D + calcium group (869 IU vs. 740 IU) [128]. During the recent years, the availability of affordable laboratory testing of the serum 25(OH)D concentrations has made it possible to have the serum 25(OH)D measured without the need to see a physician. If those in the placebo group find that their serum 25(OH)D concentrations are lower than recommended, they may increase their vitamin D intake from own supplements or from fortified foods. In addition to potentially revealing the subject his/her group and thus resulting in unblinding, this would dilute the difference in the vitamin D exposure between the experimental and control groups.
5. The supplementation period in the large trials is designed to last a maximum of 5 years. For rare diseases or for diseases with a long induction time, such as many cancers, this may be too short a time to detect benefits on incidence [238] (although the 4-year CAPS study did find a borderline statistically significant effect on total cancer incidence, even with the rather high average baseline serum 25(OH)D concentrations [128].
6. Finally, RCTS with only a few thousands of subjects may also be underpowered to detect small to moderate effects on most outcomes and, especially, to investigate whether the effects differ based on the starting serum 25(OH)D concentrations. However, pooling of the results from these RCTs will to some extent alleviate the issue of limited power in individual studies, if the serum 25(OH)D concentration data are standardized.

### Potential side effects of vitamin D

Vitamin D under selected circumstances may increase the risk of several adverse events including fractures, falls and functional decline, kidney stones, cardiovascular disease, and mortality.

#### Falls and fractures

The first indication that vitamin D could increase the risk of fracture was reported in a trial that involved supplementation with the high dose of 300,000 IU of vitamin D2 versus placebo intra­muscularly once per year for 3 years in 9,440 adults aged 75 years and older [239]. In that pragmatic trial, vitamin D had no effect on non-vertebral fractures, but it significantly increased the risk of hip fracture. This study was followed by another in which annual dosing with 500,000 IU of vitamin D3 significantly increased the number of falls and fractures in women aged 70 years and older [217]. Increased risk of falling has subsequently been observed in a trial that employed monthly dosing with 60,000 IU of vitamin D3 [198]. The comparator group in this trial received 24,000 IU per month. Two trials have tested the effect of 100,000 IU of vitamin D per month on fall risk, one in community-dwelling elders [227] and the other in nursing home residents [240]. This dose had no effect on fall risk in the community-dwelling elders but it doubled the risk of falling in the nursing home residents. The initial mean serum 25(OH)D concentrations in the two studies were similar, 60.9 and 57.4 nmol/L, respectively.

#### Kidney stones

Vitamin D in combination with supplemental calcium can, under selected circumstances, increase the risk of kidney stones. In the 7-year Women's Health Initiative (WHI), daily treatment with 400 IU of vitamin D3 plus 1,000 mg of calcium significantly increased risk of kidney stones by 17% when compared with the placebo [241]. The increased risk did not emerge until the 5^th^ year of supplementation [242]. WHI participants were allowed to continue to take their personal calcium supplements and as a result, initial mean calcium intake was over 1,100 mg per day. With supplementation, it exceeded 2 grams per day. Increased stone risk has not been seen in other trials, possibly because the other trials have been of shorter duration and have not allowed personal calcium or vitamin D supplement use. In a recent 4-year trial testing the effect of 2,000 IU of vitaminD3 plus 1,500 mg of calcium daily for 4 years on cancer risk, there were 16 kidney stones in the supplemented group and 10 in the placebo group [128].

#### Cardiovascular disease

With respect to the risk of cardiovascular disease, the large ViDA study administered 100,000 IU of vitamin D3 or placebo monthly for 3.3 years to adults age 50-84 years and reported no effect on cardiovascular disease events (primary outcome) [226]. Observational studies have noted a reverse J-shaped association of serum 25(OH)D with cardiovascular disease mortality [243]. However, a meta-analysis of 24 RCTs concluded that there was no effect of vitamin D alone on mortality but that vitamin D with calcium reduced mortality in the elderly [244].

In conclusion, high intermittent doses of vitamin D have significant potential to increase the risk of falls and fractures and are therefore an undesirable dosing regimen. Long-term use of vitamin D in combination with substantial doses of calcium (1,000 to 1,500 mg per day) has increased risk of kidney stones in participants with relatively high usual calcium intake. Prevailing evidence indicates that vitamin D supplementation does not significantly alter risk of cardiovascular disease events. Vitamin D supplementation has not been demonstrated to alter mortality and vitamin D together with calcium may reduce mortality.

**Research agenda/recommendations**

Despite our incomplete knowledge of the role of vitamin D in many target tissues, we know that serum 25(OH)D concentrations <50 nmol/L are likely to have adverse effects on health and that this affects one-quarter of the world’s population. This is a global health problem that needs to be corrected. The ideal RCTs for evaluating health benefits of vitamin D supplementation should have the following charachteristics [245–248]:

- Screening test for serum 25 (OH)D
- Inclusion of subjects based on their baseline vitamin D levels
- Ideal biochemical target according to different outcomes
- Optimal dosing regimens for vitamin D

## Abbreviations

**Acronyms (in order of appearance in text)**
25(OH)D: 25-hydroxyvitamin D
25(OH)D2: Ergocalciferol or vitamin D2
25(OH)D3: Cholecalciferol or vitamin D3
1,25(OH)2D: Calcitriol
PT/EQA: Performance testing/external quality assessment schemes
DEQAS: Vitamin D External Quality Assessment Scheme
NIST: US National Institute for Standards and Technology
VDSP: Vitamin D Standardization Program
LC-MS/MS: Liquid chromatography-tandem mass spectrometry
TUL: Tolerated upper levels
UL: Upper levels
CKD: Chronic kidney disease
CYP3A4: Cytochrome P450 3A4
HPLC: High performance liquid chromatography
RDA: Recommended Dietary Allowance
PTH: Parathyroid hormone
CAP: College of American Pathologists
UVB: Ultraviolet B
UVR: Ultraviolet radiation
CPDs: Cyclobutane pyrimidine dimers
SPF: Sun Protection Factor
UVA-PF: UVA Protection Factor
UVI: UV Index
GIO: Glucocorticoid-induced osteoporosis
SHPT: Secondary hyperparathyroidism
VDR: Vitamin D receptor
MBD: Mineral and bone disorder
K/DOQI: Kidney Disease Outcomes Quality Initiative
KDIGO: Kidney Disease: Improving Global Outcomes
TRPV6: Transient receptor potential cation channel subfamily V member 6
FGF23: Fibroblast Growth Factor 23
RCTs: Randomized controlled trials
EGFR – TKI: Epidermal growth factor receptor tyrosine kinase inhibitor
ER: Estrogen receptor
AIs: Aromatase inhibitors
SERMs: Selective receptor modulators
SERDs: Down-regulators of the ER
ID-1: Inhibitor of differentiation/DNA binding
IOM: Institute of Medicine
CD: Celiac disease
HLA: Human Leukocyte Antigen
IBD: Inflammatory bowel disease
T1DM: Type 1 diabetes mellitus
T2DM: Type 2 diabetes mellitus
BMD: Mineral bone density
TNFα: Tumor necrosis factor α
IL-6: Interleukin – 6
FPG: Fasting plasma glucose
NAFLD: Non-alcoholic fatty liver disease
FM: Fat mass
BPD: Biliopancreatic diversion
MS: Multiple sclerosis
AD: Alzheimer’s disease
IGF-1: Insulin-like growth factor 1
CHD: Coronary heart disease
VITAL: Vitamin D and Omega-3 trial
ViDA: Vitamin D Assessment
COPD: Chronic obstructive pulmonary disease
DO-HEALTH: Vitamin D3 - Omega3 - Home Exercise - Healthy Ageing and Longevity Trial
TIPS-3: The International Polycap Study 3
MACE: Major adverse cardiovascular events
D2d: Vitamin D and Type 2 Diabetes Study
WHI: Women's Health Initiative
